# Dynamic Evolution Analysis of the Emergency Collaboration Network for Compound Disasters: A Case Study Involving a Public Health Emergency and an Accident Disaster during COVID-19

**DOI:** 10.3390/healthcare10030500

**Published:** 2022-03-09

**Authors:** Jida Liu, Changqi Dong, Shi An, Qiang Mai

**Affiliations:** School of Management, Harbin Institute of Technology, Harbin 150001, China; kittadada@yeah.net (J.L.); 21b910041@stu.hit.edu.cn (C.D.); anshi@hit.edu.cn (S.A.)

**Keywords:** compound disaster, public health emergency, emergency collaboration network, COVID-19 pandemic, social network analysis, dynamic evolution

## Abstract

Compound disasters are highly complex and can involve different types of disasters. Since the beginning of the COVID-19 pandemic, compound disasters of public health emergencies, accident disasters, and natural hazards have occurred frequently all over the world; therefore, it is important to establish effective compound disaster emergency collaboration networks. Thus, this study examined the 7 March building collapse in Quanzhou City as a case study. This case was a typical compound disaster involving a public health emergency and an accident disaster during COVID-19. Based on the network analysis, the overall response and dynamic characteristics of the emergency collaboration for compound disasters were examined in this study. A compound disaster emergency collaboration network (ECN) was constructed by identifying the interactional relationships between emergency organizations. After applying time slices, the dynamic evolution of network structure, organizational–functional relations, organizational attributes, and cross-organizational relationships were discussed. The research results showed the following: (1) The density and connectivity of the compound disaster ECN first decreased before increasing. Meanwhile, the evolution of the network structure followed a path from decentralized to concentrated and from being uneven to an equilibrium. (2) The characteristics and practices of compound disasters during different periods indicated varied emergency needs for emergency organizations. We found that the formation of emergency tasks not only involved the passive adaptation to match the practice for compound disasters, but also the active choices of emergency organizations when facing compound disasters according to their collective experiences and decisions. (3) The national emergency management departments, the government emergency rescue organizations, and the local governments were the core organizations of the ECN. Public health management departments and social organizations were also required to participate in the ECN to improve the diverse and heterogeneous distribution of resources. (4) With increased demands during a compound disaster emergency, the number of cross-organizational collaborative relationships gradually increased. This study explored compound disaster emergencies from the perspective of network analysis to improve our understanding of the current and developing organizational relationships and practices during a compound disaster event. The dynamic characteristics of compound disasters require efficient adaptation and improvements of the collaborative mechanisms involved during emergencies.

## 1. Introduction

With the rapid development of society, the interactions between the natural environment and human society are constantly evolving, which can compound difficulties during disasters [1]. As a result, disasters, whether human or natural, are no longer independent, and complex, systematic emergency management is needed. The complexity of disasters can cause compound effects [2,3,4] or cascading effects [5,6]. On the one hand, compound disasters are the simultaneous occurrence of multiple types of disasters and direct influences of each other. On the other hand, cascading disasters tend to happen sequentially but within a short period; they may also be referred to as “chain disasters”. The disasters studied in this article happened concurrently and with compounded risk factors as a result of their simultaneous impacts.

Since the beginning of the COVID-19 pandemic, there have been many compound disaster scenarios that are even more complex as a result of an existing, ongoing public health emergency in addition to disasters caused by natural or human actions (e.g., droughts, earthquakes, or major vehicular accidents) [4,7]. Only one type of medical rescue team or emergency response force can not effectively respond to the emergency needs of compound disasters, so it is urgent to further coordinate and cooperate among emergency organizations [8]. In practice, strengthening the collaboration between emergency organizations, including medical treatments, rescue, and security, has become a global trend in response to disasters. These collaborative efforts between emergency organizations reduce resource waste as well as unify and improve emergency messaging and responsiveness [9,10]. These collaborative relationships have been found between governments and social organizations, central organizations and local organizations, and local organizations and neighborhood organizations [11,12].

As a novel theoretical proposition, compound disaster emergencies have attracted the scientific community, but the research has not yet caught up since it must examine the specifics and account for the differences between compound disaster emergencies [1,2,3,4]. The COVID-19 pandemic has made this especially critical when discussing and clarifying the mechanisms for collaboration between organizations during compound disasters. A network structure may be effective when responding to the complexity and uncertainty of disasters [13]. Under the networked organizational structure, emergency organizations match and unify their emergency responses through work coordination and joint decision making [14,15]. It is very appropriate and critical to introduce the network analysis method to examine and discuss the collaborative relationship in compound disasters. At present, the relevant research are focused on forming an effective organizational network for emergency responses to various disasters [16,17,18,19]. Meanwhile, previous studies also described networking interactions between functional organizations in emergency management and measured the performance of emergency management [20,21,22,23]. In addition, the frequency and complexity of disaster development and emergency action have prompted a deeper examination of the dynamics and adaptability of emergency networks [24,25].

Therefore, this study explored the characteristics of emergency collaborations during compound disasters, particularly during the ongoing, global COVID-19 pandemic by employing network analysis [26]. In particular, we examined two theoretical questions:

(1) Within the context of the COVID-19 pandemic, what are the characteristics of emergency collaboration during compound disasters?
(2) Within the context of the COVID-19 pandemic, how have emergency collaborations developed and changed in response to compound disasters? What are the dynamics of this evolution?

In detail, the above two questions need to be answered from four aspects: the structural characteristics of emergency collaboration networks, the corresponding relationships between emergency organizations and functions, the roles and the positioning of emergency organizational nodes, and the interactions between different types of emergency organizations.

The structure of this paper is organized as follows: Section 2 discusses a literature review of compound disasters and emergency networks. Section 3 details the case study and introduces the schemes of the study, as well as the indicators and parameters of the applied network analysis method. Section 4 presents the dynamic evolution of network structures, organization–function relations, organizational attributes, and cross-organizational relationships. Section 5 details the research results and clarifies their applicability. Section 6 presents the core contributions of this research and puts forward implications, limitations, and future research directions.

## 2. Literature Review

Compound disasters are formed by the coupling of two or more disasters, such as natural hazards, accident disasters, and public health emergencies. Compound disasters are characterized by multiple causal and interwoven influential factors [27]. At present, governments around the world have optimized their risk prevention, control, and disposal systems for single emergencies. However, for compound disasters, there is no effective response to solve complex problems, such as disaster chains and compounding risks [28]. As compared with the previous practices in emergency management, compound disaster emergency management intensifies the scale and expansion of the interactions and cohesion between emergency organizations [29]. Previous studies suggested that understanding and utilizing the relations among organizations are the keys to improving the efficiency of emergency responses and management [12].

In organizational relations, collaboration prevents disorder and chaos between organizations. It not only emphasizes cooperation between organizations according to common goals but also encourages their mutual support. Organizational collaboration involves sharing information and resources, or even integrating them, to resolve problems that cannot be efficiently addressed while operating independently. Meanwhile, it is an effective way to govern complex public affairs and resolve conflicts of interest or responsibility when implementing emergency management policies and responses [30]. Besides, collaboration between organizations can achieve multisectoral cooperation and coordination without eliminating organizational boundaries while effectively engaging and integrating all available resources to improve emergency responses [31,32,33].

The network analysis method was introduced in this study to explore the characteristics of organizational collaboration in emergency responses to compound disasters during the COVID-19 pandemic. In recent years, to describe the cooperation and interaction between emergency organizations, emergency networks have been extensively studied [18]. A network itself is an organizational structure with multiple agents and polymorphic nodes [14]. It has often been used to solve problems that a single organization could not solve independently [34]. For research objects, researchers have typically used actual cases of different types of disasters to study the emergency organizations’ responses to disasters, such as earthquakes, hurricanes, forest fires, snow and ice disasters, chemical accidents, and public health emergencies. For data collection, researchers have analyzed whether emergency organizations participated in disaster responses and emergency actions with other organizations based on the text data of contingency plans, news reports, and investigation reports, as well as interview and questionnaire survey data [35,36].

To meet the demands of disaster emergencies, different types, regions, and levels of emergency organizations are the basic elements of an effective emergency network. Therefore, the primary goal of this study was to analyze the organizational types of an emergency network. More diversity in the types of organizations involved typically yields better heterogeneity among the emergency network, which has shown to be an important factor when handling emergencies. Emergency networks usually comprise government organizations, private organizations, non-profit organizations, and individual volunteers. Kapucu studied the terrorist attack on 11 September 2001, in the United States and found that 73 federal agencies, 1176 nonprofit organizations, and 149 businesses were involved in the crisis and rescue responses [37]. Comfort and Hasse analyzed Hurricane Katrina’s impact on the communications infrastructure in New Orleans, Louisiana. They found that 305 public organizations, 84 nonprofit organizations, and 143 businesses were among the 535 organizations involved [38].

To explore the positions and roles of emergency organizations in the network, researchers have selected degree centrality, closeness centrality, betweenness centrality, eigenvector centrality, structural hole, and other indicators to classify emergency organizations. Comfort and Hasse analyzed the frequency of interactions between organizations in an emergency network, and they measured the effectiveness of information transmission between organizations based on closeness centrality and used betweenness centrality to determine the ability of organizations to establish connections with other organizations [38]. Zaw and Lim started with different emergency tasks and then applied in-degree and out-degree centralities to analyze the role of the military in state- and province-level disaster risk management [39].

The emergency network presents a core–periphery structure to define the roles of various emergency organizations. Nowell et al. suggested that an effective emergency network was more likely to have a core–periphery structure with moderate characteristics. Correspondingly, the study categorized emergency organizations (i.e., nodes) as key, isolated, or peripheral organizations to define their roles and positions in emergency networks [40]. Government organizations and militaries are the core organizations in a network, while non-governmental organizations are usually located at the periphery and edge of an emergency network [41]. After analyzing the emergency networks and responses to Hurricane Katrina, Curtis proposed that the effectiveness of the emergency network was affected when functional emergency organizations and responsible emergency organizations were isolated nodes or peripheral nodes [42].

The structural characteristics of emergency networks are important to identify to evaluate cooperation between emergency organizations. Meanwhile, the nature of emergency management will determine some inherent properties. Kapucu and Garayev discussed the network structure and network performance of horizontal and vertical networks in emergency management [43]. An emergency network with a vertical, hierarchical structure generally has a better system for rapid responses [11]. However, excessive centralization of a command system and administrative constraints hinder effective coordination between emergency organizations to a certain extent [44]. An emergency command system was proposed as the typical vertical, hierarchical structure. The horizontal, decentralized structure of emergency networks is derived from the spontaneous cooperation of emergency organizations during disasters [17,45]. Autonomy is the prominent feature of a horizontal, decentralized structure. In addition, an emergency organization network can also exist as a hybrid structure [46] in which the basic characteristics of vertical and horizontal structures complement each other. When analyzing network structures, researchers have typically chosen network density, network centralization, network cohesion, network clustering coefficient, average path length, and other overall network indicators for research. Correspondingly, relevant research has explored the closeness of organizational connections by measuring the clustering trends and degrees of dependence from the network to the core nodes, evaluating the connectivity of the network, considering whether the emergency organization has the characteristics that allow for dispatching, and measuring the average distance of relations between the emergency organizations.

Emergency organization networks must adapt to the dynamics and uncertainties of each disaster. Therefore, some researchers analyzed emergency networks using time slices while examining the timing of interactions between key organizations. Furthermore, a dynamic emergency network not only adjusts to the shifting demands of a disaster but also adapts to critical needs by prioritizing different organizations and their relationships as demands shift throughout the catastrophic event.

Du et al. set up an emergency response network based on a time slice of an emergency response process, specifically the hazardous chemical explosion in Xiangshui, Jiangsu Province, on 21 March 2019 [47]. Research suggested that emergency response networks tend to be decentralized over time. Lu et al. constructed an emergency communication network by taking the COVID-19 outbreak in Hubei, China, as an example [48]. The study revealed the dynamics of emergency organizations and functions in their communication networks. Abbasi and Kapucu evaluated the change in the structure and role in an emergency organization network over time using the case of Hurricane Charley. The out-degree centralization of an emergency management network was enhanced over time, and the coordination level between core emergency organizations was not continuously at a high level [49]. Further, Abbasi analyzed the link formation between emergency organizations in an emergency response network. According to the research, emergency resources and potential energy were cumulative, and the emergency organizations with stronger relationships and resources were better able to renew that relationship over time when required [50]. Htein et al. compared the differential characteristics of an emergency collaborative network during flood relief in Myanmar in 2015 and 2016, which represented a shift from being military-centered to a more integrated network involving local services and the community [51]. At present, researchers have reached a consensus on the dynamic characteristics of emergency networks; however, due to the lack of objective data, research has not yet defined the time point divisions of the dynamic development in a disaster emergency.

In summary, existing studies lack an in-depth understanding of the collaborations formed during compound disasters. Meanwhile, little research has been published regarding emergency networks during compound disasters. Given the global COVID-19 pandemic, which immediately complicates any disaster event and the responses to it, it is necessary to explore the integrations and interactions between emergency organizations. Therefore, this study considered a compound disaster case involving both a public health emergency and an accident disaster occurring in China during the COVID-19 pandemic. Furthermore, the research will deeply analyze and clarify the question, what are the characteristics of emergency collaboration, and what are the dynamics of the emergency collaborations in response to compound disasters? 

## 3. Methodology

### 3.1. Research Design and Framework Construction

**Figure 1:**
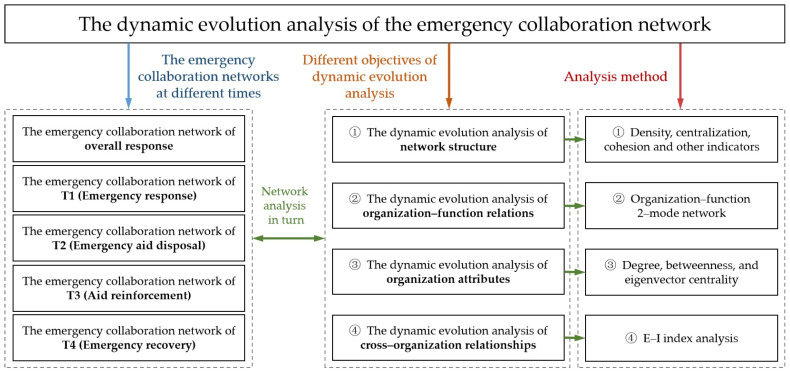
Research framework.

To answer the aforementioned research questions, this study analyzed the dynamic evolution of an emergency collaboration network (ECN) during a compound disaster. The research mainly included the following five steps, which were the theoretical framework of our research (see Figure 1). First, emergency data of typical compound disasters were collected via reports and case data. Based on the stage characteristics of compound disasters within the context of the COVID-19 pandemic, time slicing was introduced to separate emergency collaboration processes, including emergency response (T1), emergency initial disposal (T2), emergency reinforcement (T3), and emergency recovery (T4). Furthermore, the types of emergency collaboration functions (ECFs) and emergency organizations at different stages were identified. Second, the compound disaster ECN at different stages was drawn using UCINET software. The dynamic evolution processes of the network structure were analyzed based on density, centralization, cohesion, and other indicators. Third, the emergency organization–function relationships as a dual-mode network of compound disasters at different stages were developed. Based on degree centrality, the critical ECFs during different periods were analyzed and the dynamic characteristics of the relationship between organizations and functions were identified. Fourth, based on degree centrality, betweenness centrality, and eigenvector centrality, the organizational attributes during a compound disaster ECN were analyzed. Afterward, the dynamic evolution of the roles and positions of emergency organizations were clarified. Fifth, to describe the dynamic evolution characteristics of cross-organizational relationships based on E–I index analysis and chord diagrams, we discussed the directions of the collaborative relationships between different types of emergency organizations through parallel and cross-type interactions.

### 3.2. Case Background

At 19:00 on 7 March 2020, the Xinjia Hotel in Quanzhou city, Fujian Province, collapsed, causing a direct economic loss of CNY 57.94 million. After the collapse, the Ministry of Emergency Management and Fujian Province immediately launched an emergency response. Governmental organizations at all levels and relevant departments of emergency management, fire rescue, police, public health, and electrical power actively participated in the emergency rescue. At 11:00 on 12 March, the site search-and-rescue work was complete. A total of 71 trapped people had been found, of which 42 survived. This case had the following characteristics:

(1) This building collapse occurred during the COVID-19 pandemic in China in 2020. At the same time, Xinjia Hotel was serving as the centralized quarantine health observation point for COVID-19 prevention and control in Quanzhou city. The accident was considered typical of compound disasters during the COVID-19 pandemic, as it was characterized by increased difficulty in rescue, increased pressure in pandemic prevention, and prominent risks of secondary accidents, such as a secondary collapse and explosion.
(2) Similar to other compound disasters, this case was significant in scope and impact. Government official website information, media reports, and emergency information of rescue agencies at all levels were relatively complete, which could provide more complete data.
(3) The emergency process of the compound disaster involved the participation of multiple organizations. It was divided into national command organizations, emergency rescue organizations, emergency support organizations, epidemic prevention and medical organizations, and regional functional institutions. The interaction and cooperation between various emergency organizations formed a complex and intensive collaborative network, which provided a viable observation perspective for studying the emergency collaboration characteristics of compound disasters.

### 3.3. Data Collection

To ensure complete emergency data from the collapse and accurately reflect the overall organizational communication and coordination in the emergency process, this study adopted a collection method that combined internet information and case report content. First, the data of the emergency response and disposal of the compound disaster from 7 March 2020 to 13 March 2020 were collected using an Internet capture method. Based on the removal of duplicate and irrelevant data, 319 valid sources related to the collaboration of emergency organizations were included. The research collected accident survey reports and assessment data from emergency management departments. Further, we went to the Fujian Fire Rescue Corps and Quanzhou Fire Rescue Detachment to perform field research, where we interviewed emergency personnel. Based on the text data, we identified the collaborative behaviors between emergency organizations during the compound disaster.

On this basis, according to the Overall National Public Emergency Response Plan, National Production Safety Accident Disaster Response Plan, National Public Health Emergency Response Plan, and other relevant documents issued by the Chinese government, combined with the functions of the Security Commission of the State Council, the Ministry of Emergency Management, and the National Health Commission, the ECFs of compound disasters were reviewed and identified, as shown in Table 1.

### 3.4. Methods of Network Analysis

#### 3.4.1. Network Construction

This study identified the communication and interactions between emergency organizations to build a compound disaster ECN. Among them, the emergency organizations were the actors in the network, and the relationships between organizations were the network connection. Further, the study built the heterogeneous emergency organization–function relationship matrix by analyzing the corresponding relationships between emergency organizations and ECFs. Based on this, the dual-mode network of compound disaster emergency collaboration could be drawn.

#### 3.4.2. Network Structure Description and Analysis

In this study, the structural characteristics of the compound disaster ECN were analyzed, and the differences in the collaboration network at different stages were evaluated by calculating the network size, collaborative relations, network density, network centralization, network cohesion, component, network diameter, and average path length. On this basis, the dynamic evolution path and law of the compound disaster ECN were determined.

First, the study identified the overview and basic architecture of the compound disaster ECN based on network size and collaborative relations, which corresponded to the number of nodes and links of the network, respectively. When the number of nodes was high, this indicated that the number of emergency organizations participating in the emergency collaboration and the scale of the network were larger. The more links there were, the more interaction relationships were formed between the emergency organizations.

Second, network density was applied to analyze the closeness between the response organizations in the compound disaster ECN. Specifically, the network density was numerically equal to the ratio of the actual number of network connections to the theoretical maximum number of connections. In other words, the network density was related to both the links and nodes. When the network density increased, the connections, communication, and cooperation between emergency organizations in the network were closer. Conversely, the smaller the network density was, the more scattered and sparse the connections between the emergency organizations were.

Third, network centralization was used as an indicator for whether there was a core node in the network and the degree of network aggregation to the core node. In general, centralization indicators included degree centralization, betweenness centralization, and closeness centralization. We selected degree centralization to analyze the compound disaster ECN. Networks with a high degree centralization are more inclined toward the core–periphery structure, and the power of nodes in the network is more concentrated. In contrast, a network with a low degree centralization is a uniform structure, and power distribution among nodes is more average.

Fourth, the average path length, network diameter, and number of components were used to analyze the connectedness in the compound disaster ECN. Among them, the average path length represented the average communication distance between emergency organizations. The network diameter was the farthest communication distance between emergency organizations. The number of components refers to the number of subnetworks that are not interconnected. When the number of components is equal to 1, this indicates that a network is fully connected and there are no independent sub-networks.

Fifth, network cohesion was used as an indicator for the dependence between nodes based on distance. The higher the network cohesion was, the more stable the compound disaster ECN was and the more balanced the relationships between the emergency organizations were. In contrast, when the network cohesion was low, this indicated that the relationship between the emergency organizations was unstable and more easily affected by node changes.

#### 3.4.3. Node Attribute Description and Analysis

In this study, degree, betweenness, and eigenvector centralities were selected to analyze the emergency organizational nodes in the compound disaster ECN. It quantified the power and role orientation of different emergency organizations in the network.

First, degree centrality refers to the total number of nodes connected to other nodes. In directed networks, degree centrality can also be divided into in-degree and out-degree centrality. If a node has a high degree centrality, this indicates that the node is in the center of the network and has high power. Since the compound disaster ECN constructed in this study was an undirected network, degree centrality was selected to analyze the emergency organizational nodes.

Second, betweenness centrality is an indicator that measures the degree to which nodes control the network resources. When the betweenness centrality of an emergency organizational node was higher, it played a more important “intermediary” role in the network. The “intermediary” role pushed the emergency organizational node closer to the center of the network with stronger control over the other nodes. Meanwhile, betweenness centrality was used as a structural hole index to express the non-redundant connections between nodes in the network.

Third, eigenvector centrality is an indicator describing the importance of neighboring nodes to a node. In other words, eigenvector centrality was a measure of the connection quality of emergency organizational nodes. When the degree centralities of the neighboring nodes were higher, the eigenvector centrality of the emergency organizational node was higher. Specifically, high-quality connectivity meant having more powerful partners that were closer to the core. Eigenvector centrality analyzed the connection quality of the emergency organizational nodes, rather than just the quantity.

In addition, we standardized the degree, betweenness, and eigenvector centralities for comparative analysis. The values of these normalized degree, betweenness, and eigenvector centralities were obtained.

#### 3.4.4. E–I Index

The quantitative relationship in the network was divided into the relationships between factions and the relationships within factions. The relationships between factions have a greater impact on the adaptability of a network. The E–I index (external–internal index) was proposed by Krackhardt and Stern to measure the degree of cliques in a network [52]. To explore the emergency collaboration characteristics of complex disasters, this study used the E–I index to analyze the interaction between national command departments, emergency rescue organizations, emergency support organizations, epidemic prevention and medical organizations, and regional functional institutions. To do so, the cross-organizational collaborative relationships between different types of emergency organizations in compound disasters were explored. Furthermore, the identification of all emergency organizations in the overall emergency collaboration was undertaken, and the level and stability of emergency organizations to deal with complex collaboration were clearly defined.

Specifically, E–I index = (EL − IL)/(EL + IL), where EL represents the number of relationships between factions and IL represents the number of relationships within factions [53]. In fact, the value of the E–I index is also equal to the ratio of the subgroup density to the density of the whole. It follows that the inequality −1 ≤ E–I index ≤ 1 is always true. When the E–I index approaches 1, it indicates that different types of emergency organizations formed more collaborative relationships and the degree of separation between emergency organizations was small. Correspondingly, when the E–I index approaches −1, it indicates that different types of emergency organizations had more internally collaborative relationships. The same type of emergency organization tended to identify with each other and had less cross-type interaction.

## 4. Results

### 4.1. The Dynamic Evolution Analysis of the Network Structure

**Figure 2:**
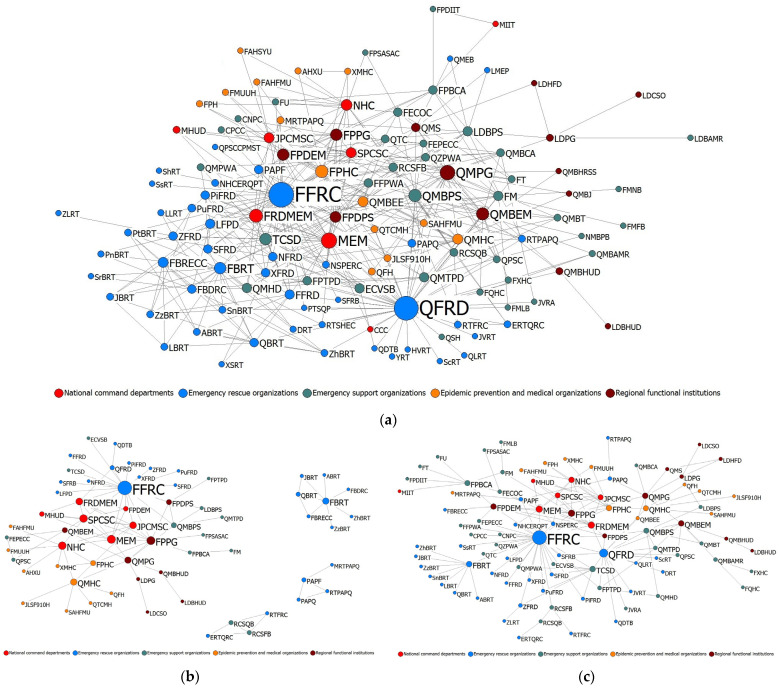

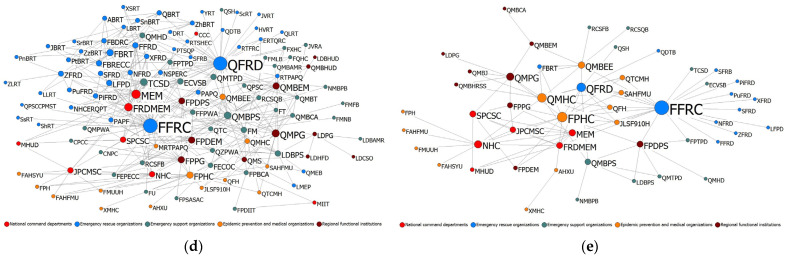
Compound disaster ECNs: (**a**) the overall response, (**b**) T1, (**c**) T2, (**d**) T3, and (**e**) T4.

To explore the dynamic evolution of the compound disaster ECN, we developed ECNs of the overall response and T1–T4 periods (in Figure 2). The organization names and abbreviations of each node in the ECNs are given in Appendix A. Furthermore, the structural indicators of the compound disaster ECNs were calculated, including the network size (nodes), collaborative relationships (links), network density, network centralization, average path length, network diameter, component, and network cohesion, as shown in Table 2.

In the compound disaster ECN overall response, 119 emergency organizational nodes were included. A total of 427 pairs of relationships were formed between the 119 emergency organizational nodes in the process of the emergency response. The network density was 6.08%, indicating that in the overall response of the compound disaster emergency collaboration, the relationships between the nodes of the emergency organization were not close, and the ECN was relatively sparse.

By comparing the characteristics of the T1–T4 periods, the network density decreased and then increased during the collaboration process of compound disasters, indicating that the degree of network concentration and the level of closeness between the emergency organizations also changed. After the transition from T1 to T2, the scale of the ECN expanded rapidly due to the participation of a large number of emergency organizations. Therefore, the density of the network was reduced to a certain extent. With the transition from T3 to T4, as the communication between emergency organizations increased and the tacit cooperation deepened, the density of the ECN increased accordingly. The contrary tendency to the density was found in the average path length and network diameter, which first increased before decreasing. This also indicated that with the influx of various emergency organizations, the interaction distance between emergency organizations increased, which reduced the overall connectivity of the network. However, with the evolution of the compound disaster emergency, the depth of coordination between emergency organizations had increased. The connectivity of the ECN and the efficiency of inter-organizational communication gradually recovered.

In addition, from T1 to T4, both the network centralization and network cohesion gradually improved. The network presented an evolution process from decentralized to concentrated and from uneven to an equilibrium. The central node of the network was clearer, and the agglomeration trend of the network to the central node was more obvious. This indicated that information and resources in the network were transferred through emergency coordination, and the resource allocation of the network was more even.

By comparing the overall response and the T1–T4 periods, the network centralization of the compound disaster ECN’s overall response was 37.77%, which was higher than those of the T1–T4 periods. This indicated that although the types of organizations involved in an emergency gradually increased, the compound disaster ECN’s overall response had the greatest tendency to move toward the central node through the exchange and cooperation of emergency organizations during different periods. Similarly, the network cohesion of the compound disaster ECN’s overall response was 0.434, which was also higher than that of the T1–T4 periods. The network diameter was 5, and the average path length was 2.576, both of which were below those of the T1–T4 periods. These results indicated that after the collaboration in response to the compound disaster emergency, a certain degree of dependency had formed between the emergency organizations in the compound disaster ECN’s overall response. Moreover, the network resources allocated by each emergency organizational node were relatively balanced; the communication distance between emergency organizations was short; and the network had high connectivity.

### 4.2. The Dynamic Evolution Analysis of the Organizational–Functional Relationships

**Figure 3:**
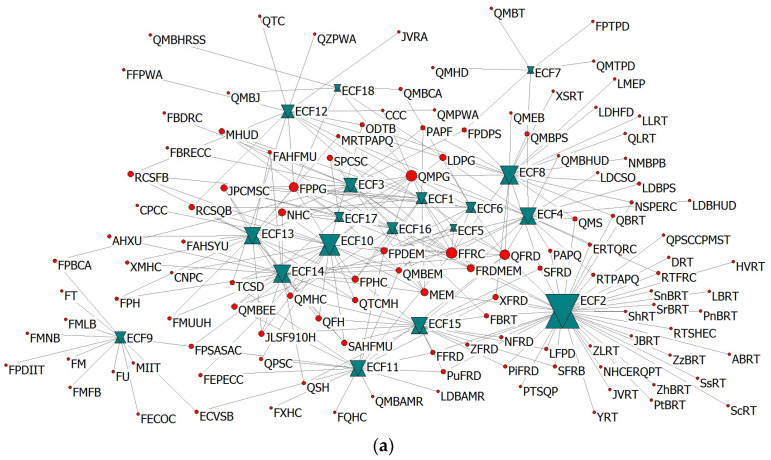

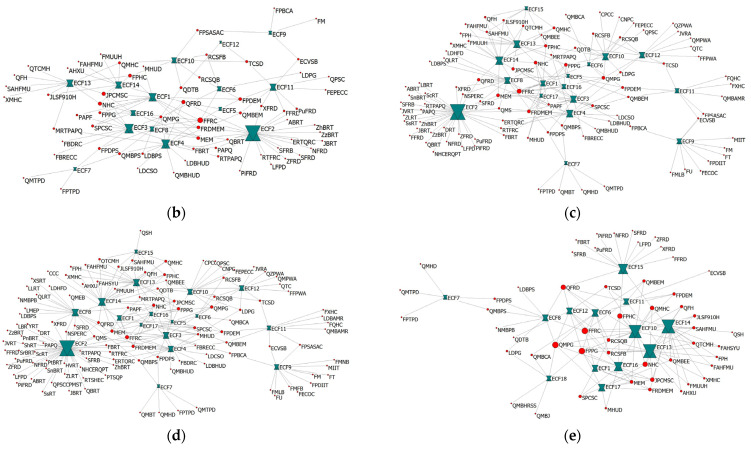
Emergency organization–function dual-mode network during the compound disaster: (**a**) the overall response, (**b**) T1, (**c**) T2, (**d**) T3, and (**e**) T4.

**Figure 4:**
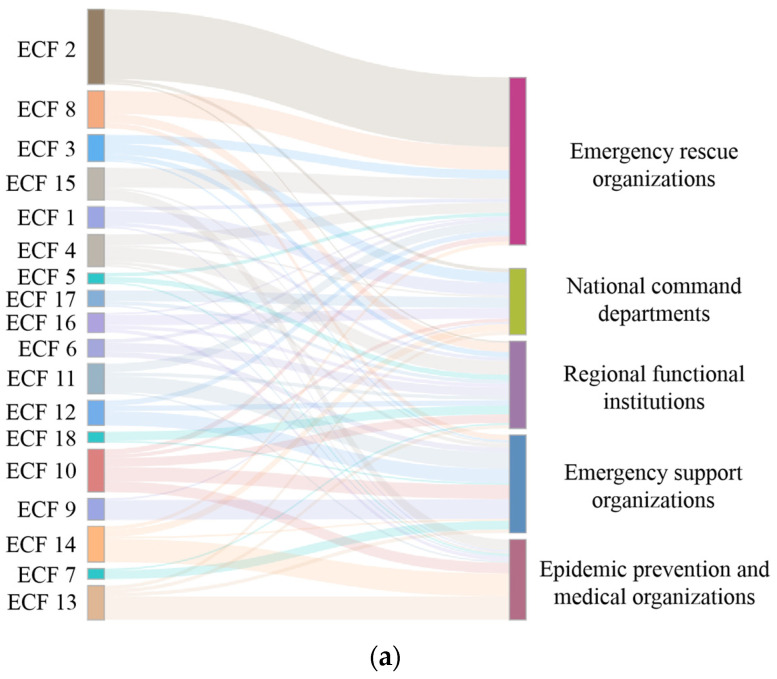

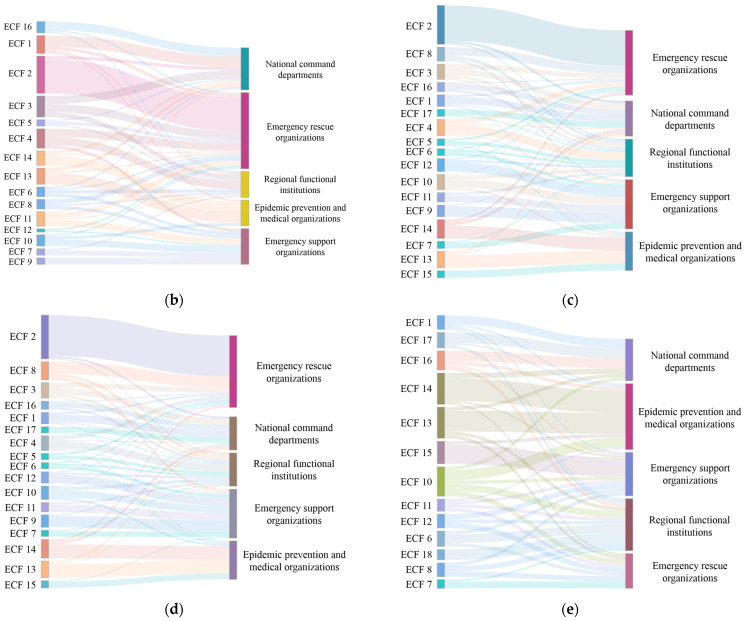
Sankey diagrams of the relationships between groups and functions: (**a**) the overall response, (**b**) T1, (**c**) T2, (**d**) T3, and (**e**) T4.

In an emergency response to a compound disaster, the collaborative relationships between emergency organizations and ECFs will change over time. To analyze the dynamic evolution of the collaborative relationships, we examined the emergency organization–function dual-mode network of the overall response and the T1–T4 periods separately, as shown in Figure 3. The organization names and abbreviations of each node in the dual-modes are given in Table 1 and Appendix A. Meanwhile, to further explore the corresponding characteristics between different types of emergency organization groups and ECFs in compound disaster emergencies, Sankey diagrams of the relationships between emergency organization groups and ECFs were drawn based on the flow relationship, as shown in Figure 4.

As shown in Figure 3, there were certain differences in the ECFs involved in the four stages of the emergency response, initial disposal, reinforcement, and recovery. From the T1 to the T4 periods, the emergency organization–function dual-mode network involved 15, 17, 17, and 13 ECFs, respectively. This suggested that in the initial disposal and reinforcement during a compound disaster emergency, emergency organizations undertook more ECFs. Therefore, there was a higher possibility of establishing connections between emergency organizations in the T2 and T3 periods. In other words, there were more opportunities for emergency collaboration between emergency organizations based on ECFs. Emergency coordination functions co-existed in T1–T4, including ECF1, ECF6, ECF7, ECF8, ECF10, ECF11, ECF12, ECF13, ECF14, and ECF16. Specifically, in the T1 period, various emergency organizations provided cooperation based on the command, personnel search, rescue, and other urgent tasks. At the same time, emergency organizations moderately expanded their functions to include support and protection, and actively deployed medical treatment and epidemic prevention teams. National command departments and emergency rescue organizations were the primary emergency organizations during this period. During the periods of T2 and T3, emergency rescue actions had become the predominant factors. To ensure the orderly and efficient implementation of emergency rescue actions, various emergency organizations focused on emergency support functions, such as providing communications, materials, and equipment. Emergency rescue and emergency support organizations utilized more ECFs during the T2 and T3 periods. In the T4 period, epidemic prevention, medical organizations, and regional functional institutions became the main organizations that performed emergency functions. Meanwhile, emergency rescue organizations also had a high participation rate during this period, as people needed to be decontaminated and quarantined.

As shown in Figure 4, the number and types of emergency organizations implementing the same ECF also evolved dynamically over time. To further identify the key role of ECFs in the process of compound-disaster emergency collaboration, we calculated the degree centrality of ECFs in a dual-mode network, as shown in Table 3. During the T1 period, the top five ECFs were ECF2, ECF3, ECF4, ECF1, and ECF13 to meet the emergency demand of a sudden and unclear disaster situation. During the periods of T2 and T3, ECF2 was still the most critical function of the compound disaster emergency. With the addition of COVID-19 risk factors, ECF13 and ECF14 were also core functions at this stage. At the same time, to match the reality of the rapid expansion of emergency organizations and resources in the emergency response, ECF8, ECF10, and other support functions also had higher degree centralities. In the T4 period, the emergency rescue and evacuation ended, and epidemic prevention, isolation, and information release were the core emergency functions. The top five ECFs were ECF13, ECF14, ECF10, ECF15, and ECF16. In summary, the task requirements in response to the compound disaster emergency showed different preferences and characteristics at different stages.

### 4.3. The Dynamic Evolution Analysis of the Organizational Attributes

To examine the dynamic evolution of the organizational attributes in the compound disaster ECN, this study calculated the normalized degree, betweenness, and eigenvector centralities of the emergency organizational nodes. Table 4, Table 5 and Table 6 present the top 10 emergency organizations in terms of their normalized centralities for the compound disaster ECN’s overall response and the T1–T4 periods.

As shown in Table 4, in the overall response of compound disaster emergency collaboration, FFRC, QFRD, MEM, QMPG, and FRDMEM had the highest degree centralities, showing that these were the most influential emergency organizations in the network. However, over time, the types of core organizations in the compound disaster ECN showed different characteristics. Specifically, in the T1 period, FFRC, FPPG, MEM, NHC, and SPCSC were the top five emergency organizations in terms of degree centrality. As government departments and provincial government departments responsible for national security, emergency management, and public health, they were significant organizations that deployed and dispatched emergency actions in the emergency response stage. Meanwhile, the compound disaster ECN was highly centralized toward them. During the T2 period, except for FFRC, FPPG, and MEM, the core positions in the network were replaced by QFRD and FRDMEM. This was due to QFRD being the emergency organization responsible for the rescue of Quanzhou City. After the accident, QFRD immediately arrived to perform the functions of emergency rescue and evacuation. FRDMEM was the emergency management department of Fujian Province. Under the command and dispatch of the national emergency management department, FRDMEM not only participated in the initial emergency action but also collected materials and emergency forces at the scene of the accident. During the T3 period, QMPG, FFRC, QFRD, MEM, and FRDMEM were the main departments responsible for emergency action. During the T4 period, with the transformation of emergency tasks, the core emergency organization combination consisted of FFRC, FPHC, QMHC, QFRD, and NHC.

By tracking the dynamic changes of the core nodes, we found that FFRC, MEM, QMPG, and FRDMEM ranked in the top ten for their degree centrality during different periods. They represented the national emergency management organization (MEM), the main emergency response force (FFRC), the territorial emergency management organization (FRDMEM), and the territorial government (QMPG), which played important roles in dispatching emergency forces and coordinating emergency resources.

As shown in Table 5, in the overall response of the compound disaster emergency collaboration, QFRD, FFRC, QMPG, FPHC, and MEM had the highest betweenness centrality and grasped more resources in the compound disaster ECN, which had significant advantages in connecting emergency organizations. Similar to the above, the types of emergency organizations that mastered network resources were different during each period.

During the T1 period, FFRC, QMPG, FPPG, NHC, and QMHC were the top five emergency organizations for betweenness centrality, which reflected the coverage of different emergency resources in the compound disaster ECN at the initial stage of the emergency. Among them, FFRC provided rescue resources; QMPG and FPPG provided institutional and administrative resources; and NHC and QMHC provided medical and epidemic-prevention resources. During the T2 period, except for FFRC and QMPG, QFRD, FBRT, and QMBEM appeared as new mesomeric organizations in the network. The above enhanced the affluence of the compound disaster ECN in terms of rescue and social resources while providing a shorter communication path for other emergency organizations. During the T3 period, FPHC and MEM, as the controllers of medical and informational resources, respectively, together with QFRD, FFRC, and QMPG, constituted the coordination node. During the T4 period, public health management departments of governments (i.e., FPHC, NHC, QMHC) were more influential bridge organizations in the network due to the change in emergency tasks.

Furthermore, to investigate the evolution trajectory of resource controllers in the network, we analyzed the top ten emergency organizations with betweenness centrality in different periods, including only FFRC and QMPG. The results indicated that the emergency organizations that controlled emergency resources and played the mesomeric role changed frequently due to the complex emergency tasks at different stages. It also reflected the fact that heterogeneous emergency response organizations provided more resources in the compound disaster ECN. The effective implementation of emergency actions required support from multiple resources.

As shown in Table 6, in the overall response of compound disaster emergency collaboration, FFRC, QFRD, MEM, FRDMEM, and QMPG had the highest eigenvector centralities. This was similar to the trend shown in Table 4. As the most influential core nodes in the compound disaster ECN, they would also have high-quality connections. It indicated that there was a “Matthew effect” in the compound disaster ECN.

During the T1 period, FFRC, MEM, SPCSC, JPCMSC, and FRDMEM were the top five emergency organizations in eigenvector centrality, and they were all national and provincial government organizations. Meanwhile, SPCSC and JPCMSC were national coordinating organizations for the safety accident and public health emergency responses, respectively. This showed that in the emergency response stage, the emergency organizations with more authority formed collaborative relationships with stronger emergency organizations that were closer to the core. During the T2 period, the emergency organizations with high eigenvector centrality were FFRC, FPPG, FRDMEM, MEM, and FPDEM. They were all emergency organizations within Fujian Province or the emergency management system, indicating that the collaborative relationships between emergency groups presented a matching effect. During the T3 period, with the advancement of emergency action, the eigenvector centrality of local emergency rescue organization QFRD and service support organization TCSD increased. Together with FFRC, MEM, and FRDMEM, they became the emergency organization combination with high-quality connections. During the T4 period, as the core controllers of medical resources, the eigenvector centralities of FPHC and QMHC increased, forming more important connections.

By tracking the dynamic changes in the eigenvector centrality of the nodes, we found that emergency organizations with high-quality connections showed a homogenization trend. At the same time, the composition of the emergency organizations also reflected the transformation from macro-emergency management departments to emergency rescue and medical treatment organizations.

### 4.4. The Dynamic Evolution Analysis of the Cross-Organizational Relationships

To analyze the resource flow and the interaction frequency between different types of emergency organizations in the compound disaster ECN, we used E–I index analysis. We also drew a chord diagram corresponding to the ECN overall response and during different periods, according to the interactions between emergency organizations. The chord diagram showed the frequency of communication between various emergency organizations. It provided a visual aid to determine the types of relationships between emergency organizations and the degree of closeness and alienation.

To test the validity of the E–I index, 5000 random permutations of the same nodes and relational links were performed. The randomness of the E–I index of the compound disaster ECN overall response and during T1–T4 periods was analyzed. Table 7 shows the observed E–I index and significance of the compound disaster ECN in the overall response and during the T1–T4 periods. The minimum, average, and maximum E–I indexes represent the results of 5000 random permutations. We found that the *p*-values of the compound disaster ECN in the overall response and during the T1–T4 periods were all equal to 0.000, indicating that each network passed the significance test of *p* < 0.001. The E–I index obtained via the analysis conducted in this study was not accidental and had statistical significance.

As shown in Table 7, the E–I index of the compound disaster ECN during T1 and T2 were −0.196 and −0.098, respectively, i.e., both were <0. This indicated that emergency organizations from different emergency groups formed more internal cooperation during the initial stage of compound disaster emergency. Different types of emergency organizations tended to form a factional emergency organizational model. In contrast, the E–I index of the compound disaster ECN during T3 and T4 were 0.005 and 0.122, respectively, i.e., both were >0. The results showed that there were more external vs. internal links between various emergency organizations during the late stage of the compound disaster emergency, which formed a strong cross-organization emergency collaboration. During periods T3 and T4, all types of emergency organizations had a higher sense of mutual identity and could efficiently manage the disaster’s evolution. Therefore, we concluded that as time passed, the cross-organizational collaborative relationships between emergency organizations in the compound disaster ECN gradually increased.

**Figure 5:**
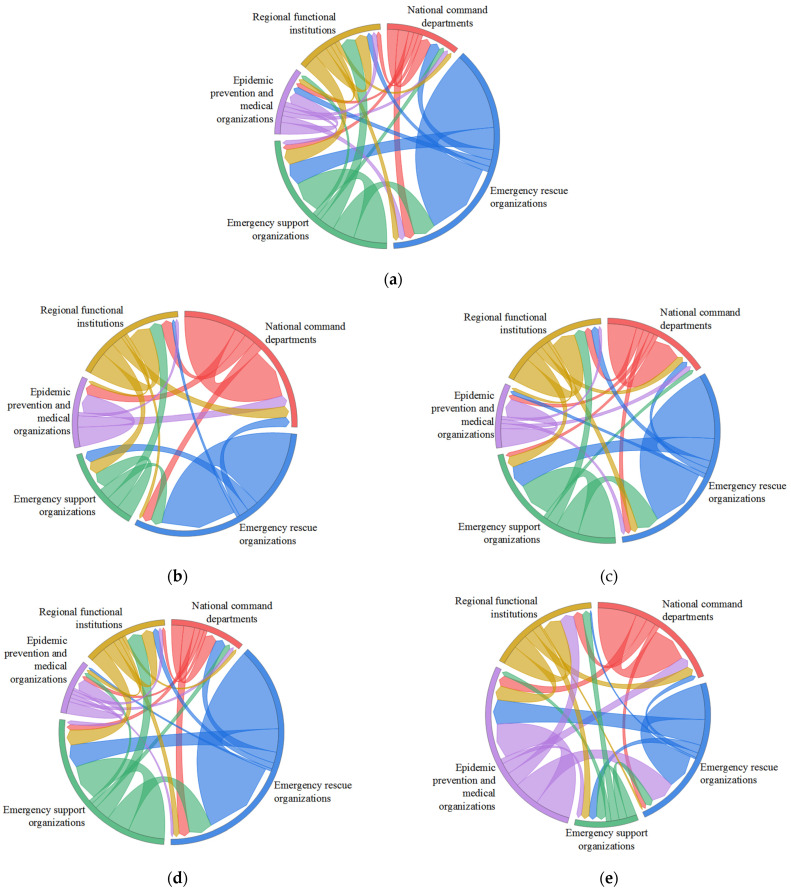
Chord diagrams of the relationships between organizational groups: (**a**) the overall response, (**b**) T1, (**c**) T2, (**d**) T3Ind (**e**) T4.

To discuss the differences in resource flows and interaction frequencies between various emergency organizations in the compound disaster ECN and analyze the dynamic evolution of cross-organizational relationships, we calculated the external/internal links and the E–I index corresponding to the overall response and during the T1–T4 periods. The calculation results are shown in Table 8 and Table 9. On this basis, the chord diagrams of emergency organization relationships corresponding to the overall response and the T1–T4 periods were drawn in turn, as shown in Figure 5.

Table 8 shows that in the overall response of the compound disaster ECN, only the E–I index of emergency rescue organizations was <0. This indicated that in the process of compound disaster emergency collaboration, it was easier to form a homogeneous collaboration between the emergency organizations that performed rescue and disposal functions, but not cross-organizational collaboration. The E–I indexes of national command departments, emergency support organizations, epidemic prevention and medical organizations, and regional functional institutions were all >0, where the values were 0.381, 0.174, 0.273, and 0.414, respectively. The results showed that the emergency organizations with command, support, rescue, and other emergency functions formed heterogeneous collaborations rather than parallel collaborations.

The E–I indexes of national command departments during the T1–T4 periods were −0.231, −0.120, 0.326, and −0.200, respectively. This indicated that national command departments conducted more horizontal emergency collaborations than vertical emergency collaborations in their emergency response, initial disposal, and recovery. Due to the dynamic and diverse nature of compound disaster emergencies, national command departments performed more emergency command actions in the emergency reinforcement phase, thus forming more cross-organizational collaboration in period T3, with an E–I index >0.

The E–I indexes of the emergency rescue organizations during the T1–T4 periods were −0.524, −0.228, −0.325, and 0.064, respectively. This indicated that the emergency rescue organizations tended to carry out parallel collaborations within rescue organizations in the stages of emergency response, initial disposal, and reinforcement. In the emergency recovery phase, emergency rescue organizations actively cooperated with emergency support organizations, epidemic prevention and medical organizations, and regional functional institutions to perform ECFs, such as epidemic prevention and elimination, isolation and resettlement, and recovery and rehabilitation, forming a series of cross-organizational collaborations. Therefore, the E–I index was >0 during the T4 period.

The E–I indexes of emergency support organizations during the T1–T4 periods were −0.200, 0.014, 0.147, and 0.368, respectively. This indicated that emergency support organizations formed positive cross-organizational collaborations with other organizations when they played auxiliary and supporting roles in the process of compound disaster emergency collaboration. Meanwhile, emergency support organizations had the highest E–I index during the T4 period, indicating that emergency support organizations played the most obvious role in promoting cross-organizational interactions in the emergency recovery stage. In contrast, during the T2 period, the E–I index of emergency support organizations approached 0. We found that the probability of intra-organizational and cross-organizational collaboration was relatively balanced for emergency support organizations at the initial disposal stage.

The E–I indexes of epidemic prevention and medical organizations for the T1–T4 periods were −0.143, −0.200, 0.077, and 0.228, respectively. This indicated that epidemic prevention and medical organizations tended to form collaborative relationships between similar organizations at the initial stage of emergency action (T1 and T2), which involved performing active deployment to deal with compound disasters in the same system and field. Comparatively, epidemic prevention and medical organizations provided epidemic prevention and treatment for command, rescue, support, and other emergency organizations in the later stage of emergency action (T3 and T4), forming a series of cross-organizational collaborations.

The E–I indexes of regional functional institutions for the T1–T4 periods were 0.152, 0.077, 0.406, and 0.273, respectively, which were all >0. This indicated that regional functional institutions tended to form collaborative relationships with other types of emergency organizations in the process of the compound disaster emergency collaboration. The corresponding E–I indexes during different periods, from high to low, were T3 (0.406), T4 (0.273), T1 (0.152), and T2 (0.077).

## 5. Discussion

With increasing disaster-causing factors, disasters may no longer appear independently. Since the beginning of the COVID-19 pandemic, compound disasters involving public health emergencies, accident disasters, and natural hazards have occurred frequently. Compound disaster emergency management has become an important issue that we need to study and understand. From the perspective of network analysis, this study examined the dynamic evolution of network structures, organizational–functional relationships, organizational attributes, and cross-organizational relationships in the process of compound disaster emergency collaboration.

(1) By analyzing the characteristics of network structure indicators during different periods, we discovered that the compound disaster ECN was dynamically evolving. On the one hand, network density and network connectivity were first reduced and then improved. After the occurrence of compound disasters, the number of emergency organizations involved in emergency response expanded. At this time, frequent interactions and communication channels between emergency organizations were yet to be established; therefore, the network density and network connectivity showed a downward trend. With the development and extension of compound disaster emergency responses, the communication and cooperation between emergency organizations for different emergency tasks increased, and the communication distance required to establish contact between emergency organizations shortened. As a result, the network density and network connectivity also increased. On the other hand, the evolution of network structures is a process from decentralized to concentrated and from being uneven to an equilibrium. As the emergency responses developed, the core nodes in the compound disaster ECN became more prominent. At the same time, resources in the network were transferred and allocated appropriately. Therefore, the compound disaster ECN gradually converged to the core organizations and tended to be balanced.

(2) In the process of compound disaster emergency collaborations, disaster characteristics and practices during different periods presented varied emergency demands for emergency organizations. Therefore, the status and level division of ECFs also presented dynamic characteristics in different emergency stages. In the initial stage of the compound disaster emergency, national and provincial emergency management departments and coordination departments implemented emergency responses centered on the core tasks of dispatching, rescue, personnel search, command, and medical treatment. After the emergency organizations arrived at the scene of the accident, the relevant emergency organizations actively expanded their functions concerning emergency rescue, and emergency support in terms of communications, materials, and equipment became the important emergency tasks. At the same time, scene alert, traffic control, and environmental monitoring as auxiliary emergency functions also appeared. When the core tasks, such as emergency rescue and on-site disposal were finished, emergency organizations prioritized different emergency tasks, such as medical treatment, epidemic prevention, disinfection, isolation, and resettlement during the emergency recovery stage. We found that the formation of emergency tasks involves not only the passive adaptation to match the practice for the compound disaster but also the active choices of emergency organizations when facing compound disasters according to their collective experiences and decisions. Passive adaptation and active selection complemented each other, which is the basic logic of the dynamic evolution in ECFs.

(3) As the compound disaster emergency required the support of diversified and heterogeneous emergency resources, the roles and positioning of emergency organizations in the compound disaster ECN were dynamic and developed at different stages. First, FFRC, MEM, QMPG, and FRDMEM were the core organizations of the compound disaster ECN and had high influence. They were the representatives of the emergency rescue force, national emergency department, local government, and local emergency department, respectively. Second, the types of emergency organizations that played the mesomeric role in the network changed frequently. Among them, the emergency organizations providing rescue resources were FFRC and QFRD. FPHC, NHC, and QMHC were the main emergency organizations connecting the medical and epidemic prevention resources. The local government QMPG and the social organization FBRT improved the resource abundance of the compound disaster ECN regarding both the institutional and social dimensions. Third, emergency organizations with authoritative and coordinating functions established high-quality connections with core nodes. With the development of emergency action, emergency rescue organizations and medical treatment organizations formed more connections with core nodes. Moreover, it was easy to establish relationships between emergency organizations with high-quality connections, which was called the “matching” effect. In addition, emergency organizations with a high degree of centrality also had high eigenvector centrality, indicating that there was a Matthew effect in the compound disaster ECN.

(4) Analyzing the differences in information flow and interaction frequency between various emergency organizations in the compound disaster ECN during different periods helped us to understand the direction and efficiency of the resource flows in the network. The implementation of emergency tasks depended on effective communication and smooth interactions between emergency organizations. More cross-organizational interactions were conducive to enhancing the sense of identity among different types of emergency organizations and improved the stability of the network. Emergency support organizations and regional functional institutions had the strongest cross-organizational collaborative relationships in different emergency stages. This was due to them effectively supporting the emergency actions of the command, rescue, and medical organizations, which provided supplies, power, communications, logistics, and other functions. National command organizations, emergency rescue organizations, and epidemic prevention and medical organizations conducted more parallel interactions between the same types of organizations. Moreover, as the complexity of the compound disaster emergency demands increased, they gradually formed more cross-organizational interactions with other emergency organizations during the middle and later stages of the emergency collaboration.

## 6. Conclusions and Implications

### 6.1. Conclusions

From the perspective of network analysis, this study examined the overall response and the evolution of time-series characteristics involved in compound disaster emergencies, particularly within the context of the COVID-19 pandemic. We identified the types and numbers of emergency organizations involved in compound disaster emergencies, and we used the 7 March Xinjia Hotel collapse in Quanzhou City as a case study, which occurred during the Chinese government’s response to the COVID-19 pandemic. Based on a social network analysis method, the compound disaster ECN and the emergency organization–function dual-mode networks were constructed by identifying the collaborative relationships between the emergency organizations. Using time slices and a combination of network indicators, Sankey diagrams, chord diagrams, and the E–I index, we discussed the dynamic evolution of network structure, organizational–functional relations, organizational attributes, and cross-organizational relationships, respectively. The results indicated the following conclusions:

(1) With the progress of compound disaster emergency actions, the density and connectivity of the compound disaster ECN first decreased and then improved. Meanwhile, the evolution of the network structure was a process that progressed from decentralized to concentrated and from uneven to equilibrium.
(2) Disaster characteristics and practices during different periods presented varied emergency demands for emergency organizations. We found that the formation of emergency tasks not only involved passive adaptation to match the practice for compound disasters but also the active choices of emergency organizations when facing compound disasters according to their collective experiences and decisions. Allocating emergency resources according to the functional orientation of each organization in the compound disaster ECN was helpful to improving the overall efficiency of the network.
(3) The emergency management departments, the government emergency rescue organizations, and the local governments were the core organizations of the compound disaster ECN. Due to the complexity of disaster-causing factors in compound disasters, public health management departments and social organizations were also required to participate in the compound disaster ECN to improve the diverse and heterogeneous distribution of the network resources. As the ECN evolved, it showed a Matthew effect and a matching effect.
(4) With the increase in the demands for compound disaster emergency action, the cross-organizational collaborative relationships between emergency organizations in the network gradually increased. Specifically, the collaborative relationships of national command departments, emergency rescue organizations, and epidemic prevention and medical organizations were transformed from parallel interactions to cross-organizational interactions. However, the collaborative relationships of emergency support organizations and regional functional institutions were usually established based on cross-organizational collaborative relationships. In general, promoting the formation of cross-organizational interactions was conducive to the improvement of network stability.

There were limitations to this study. This study examined a 7 March collapse in Quanzhou City as a case study, which represented a typical compound disaster involving a public health emergency and an accident disaster. This was a single case study, which means the results cannot be easily generalized. Further research should include a comparative study of multiple examples. The next step should also be combined with a further review of emergency practices while considering the typical compound disaster formed by the combination of public health emergencies and natural hazards for in-depth discussion. In addition, this study found that a Matthew effect and a matching effect existed in the compound disaster ECN analysis, but their generality needed further verification. Furthermore, it is necessary to introduce inferential network analysis methods, such as exponential random graph models (ERGM) and a stochastic actor-oriented model (SAOM), to verify and explain the formation characteristics and influencing factors of the compound disaster ECN.

### 6.2. Implications

From the perspective of emergency practices, further expansions on the conclusions of this research on compound disaster emergency collaboration could focus on improving emergency efficiency and optimizing the emergency mechanisms, as well as further detailing and improving the dynamic nature of these disasters and how collaboration could further be enhanced to improve adaptive efficiency.

(1) Responding to the multi-situational demands of compound disaster emergencies requires matching the dynamic characteristics of the disaster emergency response. In the process of a compound disaster emergency, both the emergency organizations and emergency functions showed dynamic characteristics, which presented a high correlation with the intensity, breadth, and type of disaster-causing factors of compound disasters. On the one hand, we need to systematically analyze the characteristics and compositions of compound disasters and identify their emergency demands and required emergency functions. Meanwhile, the key time points of compound disaster emergencies should be identified based on the differences between compound disaster scenarios, and the core emergency tasks at different stages should also be clarified. It is necessary to adjust the emergency targets and strategies according to the stages to make accurate predictions. On the other hand, we need to focus on the key role of the core emergency organizations in different emergency phases and deploy the core emergency organizations for emergency action. The emergency resources of the core emergency organizations should also be clearly defined. Furthermore, it is important to guide core organizations to realize communication and coordination with periphery organizations and provide sufficient functional space for core organizations to reduce information asymmetry and flow distortion.

(2) Responding to the proposition of compound disaster emergency management requires the improvement of mechanisms employed in cross-organizational emergency collaboration. From the compound disaster emergency collaboration’s overall response, the tightness and connectivity of the ECN still require improvements. The mechanism of cross-organizational emergency collaborations for compound disasters should be further improved to overcome the barriers impeding efficient information exchange and resource interaction. First, we should increase efforts to establish a complete, accurate, and reasonable emergency policy support system and contingency plan system according to the division of functions to improve the institutional guarantee. Meanwhile, we should adhere to the combination of normal and abnormal management systems and accelerate the professional management, the whole process management, and intensive management process of emergency management. Second, coordination mechanisms at all levels and dispatching platforms should play a bridging role in emergency management. The compound disaster emergency command structure should be consolidated to strengthen the information interaction between on-site command and rear dispatch. Furthermore, the efficiency of emergency command should be comprehensively improved. Third, we need to highlight the integrated deployment of multi-tiered emergency groups for compound disasters and increase the connection and integration of different emergency organizations. A comprehensive emergency collaboration system integrating various forms of emergency organizations should be established to avoid the disorderly pooling of emergency groups. Finally, it is equally important to realize the integrated configuration of the compound disaster emergency rescue and support system. We should not only highlight the unified dispatch of emergency equipment but also realize the stable and smooth communication network and strengthen the supply and distribution of logistics materials.

## Figures and Tables

**Table 1:** Organization ECFs.

ECF1 Dispatch and Deployment	ECF2 Emergency Rescue	ECF3 Command and Arrangement	ECF4 Personnel Evacuating
ECF5 Monitoring and warning	ECF6 Social mobilization	ECF7 Traffic control	ECF8 On-site order maintenance
ECF9 Communication support	ECF10 Material supply	ECF11 Equipment support	ECF12 Logistics subsidies
ECF13 Medical treatment	ECF14 Epidemic prevention and decontamination	ECF15 Isolation and resettlement	ECF16 Information disclosure and release
ECF17 Accident investigation	ECF18 Recovery and aftermath		

**Table 2:** Characteristic attributes of ECNs.

Indicators	Overall Process	T1	T2	T3	T4
Network size (nodes)	119	64	95	117	52
Collaborative relations (links)	427	97	153	390	98
Network density (%)	6.08	4.81	3.43	5.75	7.39
Network centralization (%)	37.77	27.80	31.27	34.50	37.18
Average path length	2.576	3.013	3.383	2.681	2.613
Network diameter	5	6	7	5	5
Components	1	4	1	1	1
Network cohesion	0.434	0.232	0.342	0.420	0.423

**Table 3:** The degree centrality of the ECFs for the overall response and during T1–T4.

ECFs	Overall Response	T1	T2	T3	T4
ECF1	12 (11)	11 (4)	10 (9)	11 (9)	8 (8)
ECF2	42 (1)	23 (1)	33 (1)	42 (1)	-
ECF3	15 (9)	13 (2)	13 (5)	15 (5)	-
ECF4	18 (6)	12 (3)	14 (3)	14 (6)	-
ECF5	6 (16)	4 (12)	6 (13)	6 (14)	-
ECF6	10 (14)	6 (10)	6 (13)	6 (14)	9 (6)
ECF7	6 (16)	4 (12)	6 (13)	6 (14)	5 (13)
ECF8	21 (3)	6 (10)	12 (7)	17 (3)	8 (8)
ECF9	12 (11)	4 (12)	10 (9)	12 (8)	-
ECF10	24 (2)	7 (8)	13 (5)	13 (7)	17 (3)
ECF11	17 (8)	9 (6)	8 (11)	9 (11)	7 (11)
ECF12	14 (10)	2 (15)	11 (8)	11 (9)	8 (8)
ECF13	19 (5)	10 (5)	14 (3)	16 (4)	18 (1)
ECF14	20 (4)	9 (6)	16 (2)	18 (2)	18 (1)
ECF15	18 (6)	-	6 (13)	7 (13)	13 (4)
ECF16	11 (13)	7 (8)	8 (11)	8 (12)	11 (5)
ECF17	9 (15)	-	6 (13)	6 (14)	9 (6)
ECF18	6 (16)	-	-	-	6 (12)

Note: numbers in parentheses represent the rank of the ECFs.

**Table 4:** The normalized degree centrality of emergency organizations in the overall response and T1–T4.

Rank	Overall Response		T1		T2		T3		T4	
1	FFRC	43.22	FFRC	31.746	FFRC	34.043	FFRC	39.655	FFRC	43.137
2	QFRD	42.373	FPPG	15.873	QFRD	17.021	QFRD	38.793	FPHC	27.451
3	MEM	22.881	MEM	15.873	MEM	11.702	MEM	21.552	QMHC	23.529
4	QMPG	20.339	NHC	14.286	FPPG	11.702	FRDMEM	18.103	QFRD	23.529
5	FRDMEM	17.797	SPCSC	14.286	FRDMEM	11.702	QMPG	16.379	NHC	17.647
6	QMBEM	16.102	JPCMSC	12.698	NHC	9.574	QMBPS	15.517	QMPG	17.647
7	FPHC	16.102	FRDMEM	12.698	FPHC	9.574	TCSD	14.655	QMBEE	15.686
8	QMBPS	16.102	QMPG	11.111	QMPG	8.511	FPDEM	13.793	MEM	15.686
9	FBRT	15.254	FBRT	11.111	FBRT	8.511	QMBEM	13.793	FPDPS	13.725
10	TCSD	14.407	QMHC	11.111	FPDEM	8.511	FPHC	12.931	FRDMEM	13.725

**Table 5:** The normalized betweenness centrality of emergency organizations in the overall response and T1–T4.

Rank	Overall Response		T1		T2		T3		T4	
1	QFRD	31.575	FFRC	27.096	FFRC	58.919	QFRD	33.05	FFRC	45.99
2	FFRC	26.162	QMPG	14.373	QFRD	30.243	FFRC	28.473	FPHC	23.252
3	QMPG	9.86	FPPG	12.04	FBRT	14.413	QMPG	9.269	NHC	15.941
4	FPHC	6.819	NHC	10.437	QMPG	13.708	FPHC	8.741	QMBEE	14.365
5	MEM	6.735	QMHC	10.041	QMBEM	12.247	MEM	6.064	QMHC	13.15
6	FBRT	5.723	MEM	8.385	FPPG	11.947	FM	5.368	QFRD	12.835
7	FM	4.713	QMBPS	5.609	FRDMEM	11.647	FPDPS	4.689	QMPG	10.686
8	QMS	3.812	SPCSC	4.968	FPDEM	10.539	FRDMEM	4.353	FPDPS	10.573
9	QMBPS	3.802	QMBEM	4.694	QMHC	9.929	QMBPS	4.312	QMBPS	7.013
10	QMBEM	3.755	JPCMSC	3.792	FPHC	9.611	FBRT	4.244	FRDMEM	5.626

**Table 6:** The normalized eigenvector centrality of emergency organizations in the overall response and T1–T4.

Rank	Overall Response		T1		T2		T3		T4	
1	FFRC	53.109	FFRC	57.226	FFRC	65.195	FFRC	54.967	FFRC	56.032
2	QFRD	45.316	MEM	51.986	FPPG	44.422	QFRD	44.074	FPHC	50.306
3	MEM	36.308	SPCSC	49.404	FRDMEM	43.571	MEM	38.146	QFRD	46.863
4	FRDMEM	30.64	JPCMSC	45.952	MEM	43.488	FRDMEM	35.101	QMHC	39.235
5	QMPG	25.767	FRDMEM	45.55	FPDEM	30.962	TCSD	29.147	FRDMEM	29.458
6	TCSD	24.933	FPPG	45.501	NHC	30.873	FPDEM	22.589	MEM	27.954
7	FPHC	22.711	FPDEM	28.949	JPCMSC	30.512	ECVSB	22.358	QMBEE	27.631
8	QMBPS	22.606	NHC	27.736	SPCSC	29.533	QMBPS	22.237	JLSF910H	26.633
9	FPDEM	21.951	MHUD	27.351	FPHC	26.367	FPPG	22.177	QFH	26.633
10	FPDPS	21.449	QMPG	20.793	QFRD	25.9	FPDPS	21.656	SAHFMU	26.633

**Table 7:** Observed E–I index with random permutations.

	Observed E–I Index	Minimum E–I Index in Permutations	Average E–I Index in Permutations	Maximum E–I Index in Permutations	Significance (*p*-Value)
Overall response	0.063 ***	0.232	0.444	0.667	0.000
T1	−0.196 ***	−0.031	0.507	0.876	0.000
T2	−0.098 ***	0.150	0.479	0.765	0.000
T3	0.005 ***	0.200	0.431	0.646	0.000
T4	0.122 ***	0.327	0.605	0.837	0.000

Note: *** *p* < 0.01.

**Table 8:** Organizational interactions in the emergency collaboration network overall response.

Organization Groups	Internal Links	External Links	Total Links	E–I Index
National command departments	30	67	97	0.381
Emergency rescue organizations	214	121	335	−0.278
Emergency support organizations	90	128	218	0.174
Epidemic prevention and medical organizations	32	56	88	0.273
Regional functional institutions	34	82	116	0.414

**Table 9:** Organizational interactions in the emergency collaborative network of T1–T4.

Organization Groups	T1 (Emergency Response)			T2 (Initial Aid Disposal)			T3 (Aid Reinforcement)			T4 (Emergency Recovery)		
	ILs	Els	E–I Index	Ils	Els	E–I Index	Ils	Els	E–I Index	Ils	Els	E–I Index
National command departments	32	20	−0.231	28	22	−0.120	30	59	0.326	24	16	−0.200
Emergency rescue organizations	48	15	−0.524	62	39	−0.228	208	106	−0.325	22	25	0.064
Emergency support organizations	10	15	0.200	36	37	0.014	90	121	0.147	6	13	0.368
Epidemic prevention and medical organizations	12	9	−0.143	18	12	−0.200	30	35	0.077	22	35	0.228
Regional functional institutions	14	19	0.152	24	28	0.077	30	71	0.406	12	21	0.273

## Data Availability

Not applicable.

## Appendix A

**Table A1:** Organizations involved in compound disaster emergency collaboration networks.

Rank	Organization Name	Abbreviation
1	Ministry of Emergency Management	MEM
2	Joint Prevention and Control Mechanism of the State Council	JPCMSC
3	Safety Production Committee of the State Council	SPCSC
4	Fujian Provincial Department of Emergency Management	FPDEM
5	Ministry of Housing and Urban-rural Development	MHUD
6	Fujian Provincial People’s Government.	FPPG
7	Quanzhou Municipal People’s Government.	QMPG
8	Quanzhou Municipal Bureau of Emergency Management	QMBEM
9	Quanzhou Municipal Bureau Administration for Market Regulation	QMBAMR
10	Quanzhou Municipal Bureau of Housing and Urban-rural Development	QMBHUD
11	Licheng District Bureau of Housing and Urban-rural Development	LDBHUD
12	Quanzhou Municipal Bureau of Civil Affairs	QMBCA
13	Licheng District People’s Government	LDPG
14	National Health Commission	NHC
15	Fujian Provincial Health Commission	FPHC
16	Xiamen Municipal Health Commission	XMHC
17	Quanzhou Municipal Health Commission	QMHC
18	Fujian Provincial Bureau of Communications Administration	FPBCA
19	Ministry of Industry and Information Technology	MIIT
20	Fujian Provincial Department of Public Security	FPDPS
21	Quanzhou Municipal Bureau of Public Security	QMBPS
22	Fujian Provincial State-owned Assets Supervision and Administration Commission	FPSASAC
23	Quanzhou Municipal Bureau of Transportation	QMBT
24	Fujian Provincial Department of Industry and Information Technology	FPDIIT
25	Licheng District Bureau Administration for Market Regulation	LDBAMR
26	Licheng District Changtai Street Office	LDCSO
27	Licheng District Bureau of Public Security	LDBPS
28	Quanzhou Municipal Highway Detachment	QMHD
29	Fujian Provincial Traffic Police Detachment	FPTPD
30	Nanan Municipal Bureau of Public Security	NMBPB
31	Quanzhou Municipal Traffic Police Detachment	QMTPD
32	Quanzhou Municipal Bureau of Ecology and Environment	QMBEE
33	Quanzhou Municipal Bureau of Human Resources and Social Security	QMBHRSS
34	Quanzhou Municipal Bureau of Justice	QMBJ
35	Fire and Rescue Department of Ministry of Emergency Management	FRDMEM
36	Fujian Fire and Rescue Corps	FFRC
37	Quanzhou Fire and Rescue Detachment	QFRD
38	Fuzhou Fire and Rescue Detachment	FFRD
39	Xiamen Fire and Rescue Detachment	XFRD
40	Zhangzhou Fire and Rescue Detachment	ZFRD
41	Putian Fire and Rescue Detachment	PuFRD
42	Longyan Fire and Rescue Detachment	LFPD
43	Sanming Fire and Rescue Detachment	SFRD
44	Ningde Fire and Rescue Detachment	NFRD
45	Pingtan Fire and Rescue Detachment	PiFRD
46	Training and Combat Support Detachment	TCSD
47	Emergency Communications and Vehicle Support Brigade	ECVSB
48	Shishi Fire and Rescue Brigade	SFRB
49	Quanzhou Detachment Training Base	QDTB
50	National Safety Production Emergency Rescue Center	NSPERC
51	National Hazardous Chemicals Emergency Rescue Quanzhou Petrochemical Team	NHCERQPT
52	Professional Team of Sinochem Quanzhou Petrochemical	PTSQP
53	Quanzhou Power Supply Company Communist Party Member Service Team	QPSCCPMST
54	Rescue Team of the Second Harbor Engineering Company	RTSHEC
55	Rescue Team of Fujian Red Cross	RTFRC
56	Emergency Rescue Team of Quanzhou Red Cross	ERTQRC
57	Red Cross Society of Quanzhou Branch	RCSQB
58	Red Cross Society of Fujian Branch	RCSFB
59	Jinjiang Volunteer Rescue Team	JVRT
60	Shishi Rescue Team	SsRT
61	Jinjiang Voluntary Rescue Association	JVRA
62	Zhangzhou Longxi Rescue Team	ZLRT
63	Fujian Bluesky Rescue Team	FBRT
64	Fujian Bluesky Rescue Emergency Coordination Center	FBRECC
65	Quanzhou Bluesky Rescue Team	QBRT
66	Jinjiang Bluesky Rescue Team	JBRT
67	Zhangzhou Bluesky Rescue Team	ZzBRT
68	Zhenghe Bluesky Rescue Team	ZhBRT
69	Anxi Bluesky Rescue Team	ABRT
70	Shouning Bluesky Rescue Team	SnBRT
71	Liannan Bluesky Rescue Team	LBRT
72	Fujian Bluesky Disaster Relief Center	FBDRC
73	Pingnan Bluesky Rescue Team	PnBRT
74	Pingtan Bluesky Rescue Team	PtBRT
75	Shangrao Bluesky Rescue Team	SrBRT
76	Fujian Quanneng Hoisting Company	FQHC
77	Fujian Xinsheng Hoisting Company	FXHC
78	Quanzhou Tzu Chi	QTC
79	Fujian Fulao Public Welfare Association	FFPWA
80	Quanzhou Zhenqing Public Welfare Association	QZPWA
81	Yangguang Rescue Team	YRT
82	Huangye Voluntary Rrescue Team	HVRT
83	Sihuang Rescue Team	ShRT
84	Dongwang Rescue Team	DRT
85	Shangcun Rescue Team	ScRT
86	Luoyuan Lanbao Rescue Team	LLRT
87	Quanzhou Leiting Rescue Team	QLRT
88	Quanzhou Micro Public Welfare Association	QMPWA
89	Xiamen Shuguang Rescue Team	XSRT
90	Quanzhou Sunjiang Hotel	QSH
91	JLSF 910 Hospital	JLSF910H
92	Quanzhou Military Sub-region	QMS
93	Licheng District Human Forces Department	LDHFD
94	Quanzhou Militia Emergency Battalion	QMEB
95	Licheng Militia Emergency Platoon	LMEP
96	People’s Armed Police of Fujian	PAPF
97	People’s Armed Police of Quanzhou	PAPQ
98	Rescue Team of People’s Armed Police of Quanzhou	RTPAPQ
99	Medical Rescue Team of People’s Armed Police of Quanzhou	MRTPAPQ
100	China Petroleum and Chemical Corporation	CPCC
101	China National Petroleum Corporation	CNPC
102	Fujian Mobile	FM
103	Fujian Telecom	FT
104	Fujian Mobile of Licheng Branch	FMLB
105	Fujian Mobile of Nanan Branch	FMNB
106	Fujian Mobile of Fengze Branch	FMFB
107	Fujian Unicom	FU
108	Fujian Emergency Communication Operation Company	FECOC
109	Quanzhou Power Supply Company	QPSC
110	Fujian Electric Power Emergency Command Center	FEPECC
111	China Communications Construction	CCC
112	Quanzhou First Hospital	QFH
113	The Second Affiliated Hospital of Fujian Medical University	SAHFMU
114	Quanzhou Traditional Chinese Medicine Hospital	QTCMH
115	Fujian Medical University Union Hospital	FMUUH
116	The First Affiliated Hospital of Fujian Medical University	FAHFMU
117	Fujian Provincial Hospital	FPH
118	The First Affiliated Hospital, Sun Yat-sen University	FAHSYU
119	The First Affiliated Hospital of Xiamen University	AHXU
